# Albuminuria and Diabetic Retinopathy in Type 2 Diabetes Mellitus *Sankara Nethralaya Diabetic Retinopathy Epidemiology And Molecular Genetic Study (SN-DREAMS, report 12)*

**DOI:** 10.1186/1758-5996-3-9

**Published:** 2011-05-25

**Authors:** Padmaja K Rani, Rajiv Raman, Aditi Gupta, Swakshyar S Pal, Vaitheeswaran Kulothungan, Tarun Sharma

**Affiliations:** 1 Shri Bhagwan Mahavir Vitreoretinal Services, 18, College Road, Sankara Nethralaya, Chennai-600 006, Tamil Nadu, India; 2 Department of Preventive Ophthalmology (Epidemiology and Biostatistics), 18, College Road, Sankara Nethralaya, Chennai-600 006, Tamil Nadu, India

**Keywords:** Diabetic Retinopathy, Microalbuminuria, Macroalbuminuria, Risk factor, Type 2 Diabetes

## Abstract

**Background:**

The concordance of microalbuminuria and diabetic retinopathy (DR) has been well reported in persons with type 1 diabetes; however, for type 2 diabetes, there is paucity of data especially from population-based studies. The aim of this study was to estimate the prevalence of albuminuria (micro - and macroalbuminuria) among persons with type 2 diabetes and determine its role as a risk factor for presence and severity of DR.

**Methods:**

A population-based cross sectional study was conducted in cohort of 1414 subjects with type 2 diabetes from Chennai metropolis. All the subjects underwent comprehensive eye examination including 45 degrees four-field stereoscopic digital photography. DR was clinically graded using Early Treatment Diabetic Retinopathy Study scales. A morning urine sample was tested for albuminuria. Subjects were considered to have microalbuminuria, if the urinary albumin excretion was between 30 and 300 mg/24 hours, and macroalbuminuria at more than 300 mg/24 hours. The statistical software used was SPSS for Windows, Chicago, IL. Student t-test for comparing continuous variables, and *χ*^2 ^test, to compare proportions amongst groups were used.

**Results:**

The prevalence of microalbuminuria in the study subjects was 15.9% (226/1414), and that of macroalbuminuria, 2.7% (38/1414). Individuals with macroalbuminuria in comparison to micro- or normoalbuminuria showed a greater prevalence of DR (60.5% vs. 31.0% vs. 14.1%, p < 0.001), and also a greater severity of the disease (60.9% vs. 21.4 vs. 9.9, p < 0.001).

**Conclusions:**

Every 6^th ^individual in the population of type 2 diabetes is likely to have albuminuria. Subjects with microalbuminuria were around 2 times as likely to have DR as those without microalbuminuria, and this risk became almost 6 times in the presence of macroalbuminuria.

## Background

In the year 2000, there were around 171 million people with diabetes globally, and by 2030, it is estimated that this number would increase to 366 million [1]. As the number of persons with diabetes increases, the development of microvascular complications like retinopathy, nephropathy and neuropathy also rises. These microvascular complications are linked to the duration of diabetes mellitus, poor glycemic control and systolic hypertension [2]. The magnitude of damage caused by these microvascular complications of diabetes stresses the need for sensitive markers of screening for retinopathy and nephropathy. The sensitive marker for the detection of diabetic nephropathy is to estimate excretion of microalbumin in urine; and for the detection of diabetic retinopathy (DR), to have a fundus evaluation after pupillary dilatation [3,4].

According to a report by the World Health Organization (WHO), the prevalence rates of nephropathy after 15 years of diabetes ranged between 17.7 and 56.6% in men and between 11.9 and 71% in women. DR is responsible for 4.8% of the 37 million cases of blindness throughout the world [5]. The concordance of microalbuminuria and DR has been well reported in persons with type 1 diabetes [6-8]; however, for type 2 diabetes, there is paucity of data especially from population-based studies regarding the association of microalbuminuria with DR [9-14]. The present population based study was carried out to estimate the prevalence of albuminuria (micro- and macro-) in type 2 diabetes mellitus and report its influence as a risk factor for the presence and severity of DR.

## Materials and methods

Study subjects were recruited from the Sankara Nethralaya Diabetic Retinopathy Epidemiology and Molecular Genetic Study (SN-DREAMS), which was a population-based cross sectional study. The study design and research methodology of SN-DREAMS 1 is described in detail elsewhere [15]. The study area was Chennai metropolis with a population of 4.3 million, distributed in 155 divisions of ten zones.

A computed sample size of 5830 individuals was estimated assuming the prevalence of DR in the general population to be 1.3%, with a relative precision of 25%, a drop-out rate of 20%, and a design effect of 2. Inclusion criteria included Individuals aged 40 years or above or turning 40 in the current year and residing for a minimum of six months at the same residence. Individuals residing for a period of less than six months at the same residence, temporary residents (whose permanent residence is elsewhere), a resident who dies after the enumeration but prior to examination, and those who cannot be contacted after five attempts by the social worker at their residence were excluded from the study.

Thus, 5999 subjects from the general population aged >40 years were enumerated. To ensure that data is collected from all socioeconomic groups, multistage random sampling was stratified, on the basis of economic criteria. To avoid patient selection bias, sampling was done in two stages: selection of divisions and selection of study subjects. Selection of divisions was done using computer generated random numbers; ensuring that one division per one corporate zone is represented in the sample. Eligible study subjects were then randomly selected from each division.

Of the 5999 subjects enumerated, 1414 persons identified with diabetes (both known and newly diagnosed) were analyzed for the study (96.20% response rate for first fasting blood sugar estimation, 85.60% response rate for base hospital examination, 8.7% turned out as non diabetic after second blood sugar and 0.78% of retinal images were un-gradable). Subjects with type 2 diabetes were identified based on the American Diabetes Association criteria [16]. Persons with known diabetes were previously diagnosed cases of diabetes whether or not they were using either oral anti-glycemic drugs or insulin or both. Persons with newly-diagnosed diabetes were those who had their fasting blood glucose level ≥110 mg/dl on two separate days. The study was approved by the Institutional Review Board, and written informed consent was obtained from subjects as per the Helsinki declaration. In order to ensure that the diagnostic measurements of glucose were quality assured, many quality control measures were taken. A comprehensive instruction manual and a start-up training session helped to standardize all the examination and diagnostic procedures. The glucometer was calibrated every day and its reproducibility was assessed by measuring the blood glucose for the same patient six times and also with two machines. The collected data was scrutinized manually before its entry into the computer. The measurements were centralized at the base hospital.

Albuminuria estimation was done by a semi-quantitative procedure (Bayer Clinitek 50 Urine Chemistry Analyzer) with the first morning urine sample [17]. The Clinitek Microalbumin strip contains two reagent areas that test for albumin and creatinine in urine and provide semiquantitative results. In this study, only the albumin results were used. The albumin test is based on dye binding, using a high-affinity sulfonephthalein dye. At a constant pH, the development of any blue color is due to the presence of albumin. The resulting strip color ranges from pale green to aqua blue. The results were read with the use of the Clinitek 50 Urine Chemistry Analyzer (Bayer Health- Care, Elkhart, Ind., USA). Sensitivity and specificity for microalbuminuria diagnosis have been reported to be 100 and 81% for Clinitek Microalbumin [18]. The diagnostic performance of measuring UAC in a spot morning urine sample in predicting microalbuminuria in subsequent 24-hour urine collections has been reported to be satisfactory, and, moreover, comparable to that of measuring ACR [19]. Subjects were considered to have microalbuminuria, if the Urinary Albumin Excretion (UAE) was between 30 and 300 mg/24 hours, and macroalbuminuria at more than 300 mg/24 hours [20]. Glycosylated hemoglobin (HbA1c) fractions were estimated by using Merck Micro Lab 120 semi-automated analyzer (Bio-Rad DiaSTAT HbA1c Reagent Kit) [16]. Severity of DR was clinically graded using Early Treatment Diabetic Retinopathy Study scales [21]. Retinal photographs were taken after pupillary dilatation (Carl Zeiss Fundus Camera, Visucamlite, and Jena, Germany); all patients underwent 45° four-field stereoscopic digital photography. For those who showed evidence of any DR, additional 30° seven-field stereo digital pairs were taken. All photographs were graded by two independent observers in a masked fashion; the grading agreement was good (k = 0.83). When the evaluation of the two independent observers of DR did not agree, a third observer was referred to.

Statistical analyses were performed using the statistical software (SPSS for Windows, ver.13.0 SPSS Science, Chicago, IL). The results were expressed as mean ± SD if the variables were continuous, and as percentage, if categorical. Student t-test for comparing continuous variables, and *χ*^2 ^test, to compare proportions amongst groups were used. Individuals with newly-diagnosed diabetes were given a value of zero for duration of diabetes. P value of ≤0.05 was considered as significant. Both univariate and multivariate logistic regression analyses were performed to study the effect of various risk factors using microalbuminuria and macroalbuminuria as dependent variables. From the univariate analysis, variables with p values ≤0.05 and those, which were already established as risk factors, were included in the multivariate logistic regression analysis to derive at the parsimonious model. These variables included age, duration of diabetes, body mass index, systolic and diastolic blood pressure, and HbA1c.

## Results

Table 1 provides the definitions of the terms used in the study.

**Table 1:** Definitions used in the Study

Sno	Variable	Definition
**1**	Albuminuria	The patient was considered to have normoalbuminuria, if Urinary Albumin Excretion (UAE) was < 30 mg/24 hour; microalbuminuria, if UAE was 30-300 mg/24 hours, and macroalbuminuria if UAE was >300 mg/24 hours

**2**	Diabetes Mellitus	Persons with known diabetes were those who were using either oral anti-glycemic drugs or insulin or both. Persons with newly-diagnosed diabetes were those who had their fasting blood glucose level ≥110 mg/dl on two separate days.

**3**	Non-sight threatening Diabetic Retinopathy	Non-sight threatening diabetic retinopathy included cases of mild or moderate non-proliferative diabetic retinopathy.^21^

**4**	Sight-threatening Diabetic Retinopathy	Sight-threatening diabetic retinopathy (Referable diabetic retinopathy) was defined as severe non-proliferative diabetic retinopathy, proliferative diabetic retinopathy and clinically significant macular edema.^21^

Table 2 shows the prevalence of micro - and macroalbuminuria in the study population. Out of 1414 subjects, 1166 were known cases of diabetes and 248 were having newly diagnosed diabetes. Overall, the prevalence of microalbuminuria in the study subjects was 226 out of 1414, 15.9% (95% CI: 13.9-17.8), and that of macroalbuminuria was 38 out of 1414, 2.7% (95% CI: 1.8-3.5). The prevalence of microalbuminuria was 191 out of 1166, 16.4% in individuals with known diabetes, and 35 out of 248, 14.1%, in those with newly diagnosed diabetes (p = 0.38). However, the prevalence of macroalbuminuria showed statistically significant differences between these 2 groups (3.1% vs. 0.8%, p = 0.04).

**Table 2:** Prevalence of Albuminuria in the Study Population.

Groups	Overall (N = 1414)	KD* (N = 1166)	NDD^† ^(N = 248)	*p *for KD vs NDD
	n (%) (95% CI)	n (%) (95% CI)	n (%) (95% CI)	
Normoalbuminuria	1150 (81.3%) (79.3 - 83.3)	939 (80.5%) (78.2- 82.8)	211 (85.1%) (80.5-9.5)	0.095
Microalbuminuria	226 (15.9%) (13.9-17.8)	191 (16.4%) (4.2- 8.5)	35 (14.1%) (9.7-18.4)	0.376
Macroalbuminuria	38 (2.7%) (1.8-3.5)	36 (3.1%) (2.1- 4.0)	2 (0.8%) (-0.3-1.9)	0.044

* KD, Persons with known diabetes; ^† ^NDD, Persons with newly diagnosed diabetes

Table 3 summarizes the clinical characteristics among the study population with regard to normo -, micro - and macroalbuminuria. The mean age of the total study population (n = 1414) was 56.3 ± 10 yrs; 750 (53.04%) were men. Several clinical parameters showed highest value in individuals with macroalbuminuria followed by micro - and normoalbuminuria. These parameters included age, duration of diabetes and systolic blood pressure (p < 0.001). The body mass index (BMI) (Kg/m^2^) was the lowest in individuals with macroalbuminuria compared to micro - and normoalbuminuria (23.5 ± 3.8 vs. 24.9 ± 4.5 vs. 25.5 ± 3.9, p = 004). The diastolic blood pressures were higher in those individuals with macro - or microalbuminuria compared to those with normoalbuminuria (83.8 ± 12.9 vs. 84.0 ± 11.5 vs. 81.5 ± 11.2, p = 005), and so was true for HbA_1c _(9.0 ± 2.3 vs. 9.0 ± 2.3 vs. 8.0 ± 2.1, p < 0.001). Individuals with macroalbuminuria in comparison to micro - or normoalbuminuria showed a greater prevalence of DR (60.5% vs. 31.0% vs. 14.1%, p < 0.001), and also a greater severity of the disease (60.9% vs. 21.4 vs. 9.9, p < 0.001). There was no significant association between serum lipids and albuminuria (p = 0.299 for total cholesterol, p = 0.119 for HDL and p = 0.896 for triglycerides).

**Table 3:** Clinical Characteristics of the Study Subjects.

Variables*	Normoalbuminuria n = 1150	Microalbuminuria n = 226	Macroalbuminuria n = 38	*p*
Male	609 (53.0)	118 (52.2)	23 (60.5)	0.631
Age (years)	55.7 ± 10.0	58.6 ± 9.6	59.6 ± 9.67	< 0.001
Duration of DM (years)	5.2 ± 6.1	6.6 ± 6.6	8.9 ± 6.2	< 0.001
BMI (kg/m2)	25.5 ± 3.9	24.9 ± 4.5	23.5 ± 3.8	0.004
SBP (mm of Hg)	137.4 ± 19.9	145.6 ± 22.8	150.4 ± 23.0	< 0.001
DBP (mm of Hg)	81.5 ± 11.2	84.0 ± 11.5	83.8 ± 12.9	0.005
HbA1c (gm %)	8.0 ± 2.1	9.0 ± 2.3	9.0 ± 2.3	< 0.001
Serum TC	186.1 ± 40.4	186.8 ± 39.3	196.5 ± 56.9	0.299
Serum HDL	39.2 ± 10.2	38.8 ± 9.3	42.5 ± 13.2	0.119
Serum TGL	153.4 ± 104.4	156.1 ± 80.4	148.9 ± 75.2	0.896
Presence of DR	162 (14.1)	70 (31.0)	23 (60.5)	< 0.001
Presence of Sight threatening DR	16 (9.9)	15 (21.4)	14 (60.9)	< 0.001

Abbreviations: DM, Diabetes Mellitus; BMI, Body Mass Index; SBP, Systolic Blood Pressure; DBP, Diastolic Blood Pressure; HbA1c, Glycosylated Hemoglobin; DR, Diabetic Retinopathy

* Data are means ± SD or n (%)

Results of regression analysis of risk factors related to albuminuria are shown in Table 4. For microalbuminuria, the significant variables included increase in age per year Odds Ratio (OR) 1.01 (95% CI: 1.00-1.04), increase in systolic blood pressure per mm of Hg OR 1.01 (95% CI: 1.00-1.02), increase in HbA1c per gm% OR 1.18 (95% CI: 1.11-1.26), presence of DR OR 2.10 (95% CI: 1.46-3.01) and sight-threatening DR OR 2.49 (95% CI: 1.15-5.37). For macroalbuminuria, significant variables included increase in systolic blood pressure per mm of Hg OR 1.02 (95% CI: 1.00-1.04), presence of DR OR 6.50 (95% CI: 3.11- 13.61), and sight-threatening DR OR 14.19 (95% CI: 5.31-37.96). Decreased BMI was associated with macroalbuminuria OR 0.89 (95% CI: 0.80-0.98).

**Table 4:** Multiple Logistic Regression Analysis of Risk Factors for Albuminuria

Variables	Microalbuminuria		Macroalbuminuria	
	Odds ratio (95% CI)	p*	Odds ratio (95% CI)	p*
				
Age (per year)	1.02 (1.00 - 1.04)	0.005	1.02 (0.98 -1.06)	0.280
Duration of DM ((per year)	1.00 (0.97 -1.02)	0.869	1.02 (0.97 - 1.07)	0.358
BMI (per Kg/m^2^)	0.97 (0.93-1.01)	0.207	0.89 (0.80-0.98)	0.028
SBP (per mm of Hg)	1.01 (1.00-1.02)	0.008	1.02 (1.00- 1.04)	0.014
DBP (per mm of Hg)	1.01 (0.99-1.03)	0.180	1.00 (0.97-1.04)	0.817
HbA1c (per gm %)	1.18 (1.11-1.26)	< 0.001	1.07 (0.92-1.25)	0.352
Presence of DR	2.10 (1.46-3.01)	< 0.001	6.50 (3.11-13.61)	< 0.001
Presence of Sight threatening DR	2.49 (1.15-5.37)	0.02	14.19 (5.31-37.96)	< 0.001

Abbreviations: DM, Diabetes Mellitus; BMI, Body Mass Index; SBP, Systolic Blood Pressure; DBP, Diastolic Blood Pressure; HbA1c, Glycosylated Hemoglobin; DR, Diabetic Retinopathy

*Adjusted for age, duration of diabetes, body mass index, SBP, DBP and HbA1c

## Discussion

The present study primarily reports the prevalence of albuminuria (micro - and macro -) amongst persons with type 2 diabetes and evaluates its role as a risk factor for presence and severity of DR. Besides, it also reports the risk factors related to albuminuria as its secondary outcome measures.

In the present study, the age- and gender adjusted prevalence of diabetes mellitus in a population older than 40 years was 28.2%. Other studies have also shown a similar high prevalence of diabetes mellitus from India [22-24].

The National Urban Diabetes Study (2000) [25] showed the prevalence of diabetes in a population older than 40 years to be 23.8% in 6 cities in India including Chennai, and more recently, the Chennai Urban Rural Epidemiology Study (2003-2004) estimated the prevalence in those older than 40 years to be 30.1% [26].

In the present study, individuals with type 2 diabetes showed prevalence of microalbuminuria to be around 16%, and that of macroalbuminuria, around 3%. It means that every 6^th ^individual in the population of individuals with diabetes had albuminuria. While occurrence of microalbuminuria was observed in equal proportions in individuals with known diabetes vs. newly diagnosed (16.4% vs. 14/1%), macroalbuminuria was more common in the former group (3.1% vs. 0.8%). The prevalence of micro - and macroalbuminuria was reported to be around 27% and 5% by Unnikrishnan *et al *[2]. Like the results of our study, they also noted that individuals with known diabetes had higher prevalence of macroalbuminuria than those with newly diagnosed diabetes (6.5% vs 3.9%); no differences were noted with regard to microalbuminuria.

Our study highlighted that subjects with microalbuminuria were around 2 times as likely to have DR as those without microalbuminuria, and this risk became almost 6 times in the presence of macroalbuminuria. A similar trend was noted for sight-threatening DR, the odds were 2.5 times for microalbuminuria and 14 times for macroalbuminuria. The DCCT in type 1 diabetes mellitus reported that there is a relationship between DR and diabetic nephropathy [27]. Within the study group that showed evidence of minimal DR at baseline, 10% had elevated UAE rates. For the population with type 2 diabetes, though the association between advanced degrees of DR and macroalbuminuria (or proteinuria) is well-known [5,13,14,28,29], the relationship with lower levels of UAE within the range of microalbuminuria is controversial. Some investigators have reported a positive association [5,28,30-33, while in other studies this has not been observed [11,34-36]. Boelter *et al *[33] also reported the presence of renal involvement, including urinary albumin excretion within the microalbuminuria range in type 2 diabetic patients with proliferative diabetic retinopathy. They emphasized that all patients with proliferative diabetic retinopathy should undergo an evaluation of renal function including urinary albumin measurements. A few studies have identified that the renal changes seen in individuals with both microalbuminuria and retinopathy had a distinct pattern compared to those having microalbuminuria without retinopathy. Severe retinopathy has been described as more closely associated with glomerulosclerosis, with Kimmelstiel-Wilson nodules, than with mesangial sclerosis [37].

Logistic regression analysis identified increasing age, raised blood pressure, and poor glycemic control as influencing microalbuminuria. Though a very subtle connection has been reported between urinary albumin, DR and lipids [38], we did not find a significant association between serum lipids and albuminuria in our study. BMI was inversely related to macroalbuminuria, however, this finding may only reflect the worst nutritional state of patients with more advanced renal disease and not a causal relationship. A poor nutritional status has been reported among subjects with diabetic nephropathy due to many factors, including higher resting energy expenditure, increased muscle protein breakdown [as reported in patients with type 2 DM undergoing hemodialysis] and, in some cases, restrictive dietary advice [39]. Raised blood pressure also influenced macroalbuminuria. The EURODIAB study in type 1 diabetes mellitus showed that the correlation between increasing blood pressure and albumin excretion rate was only confirmed in patients who also had DR, independently of glycemic control or diabetes duration, suggesting that DR, in association with increased blood pressure, is an important independent risk factor for diabetic nephropathy progression [40]. Unnikrishnan *et al *[2] reported association of systolic blood pressure with both micro- and macroalbuminuric groups and diastolic blood pressure with only microalbuminuric group. Poor glycemic control as a risk factor for microalbuminuria was reported in various studies [2,5,7]. Good glycemic and hypertension control should be recommended to prevent the occurrence of these complications of diabetes.

The association between microalbuminuria and DR observed in the present study could be explained by the view that microalbuminuria might represent a state of generalized vascular dysfunction [41]. Enzymes involved in the metabolism of anionic components of the extracellular matrix (e.g. heparan sulphate proteoglycan) vulnerable to hyperglycaemia, seem to constitute the primary cause of albuminuria and its associated complications. Genetic polymorphism of such enzymes [41], as well as of several candidate genes [42-47] has been hypothesized to be the main reason for the variation in susceptibility.

Alternatively, microalbuminuria and DR may share common risk factors. The duration of diabetes and blood pressure levels are well-known risk factors for both DR and diabetic nephropathy [48,49]. There are other factors which damage vessels in both retina and kidney. For example, Klein *et al *[50] showed that microalbuminuria could be seen in 29.2% of insulin taking patients and 22% of non-insulin dependent patients. Likewise, Kim *et al *[51] reported that fasting plasma level of insulin and systolic blood pressure has independent correlation with microalbuminuria. Besides common mechanisms, renal damage may accelerate retinopathy which is associated with serum levels of fibrinogen and lipoproteins [52]. Also, albuminuria has been considered as a predictor of DR and coronary heart disease [53].

There are potential shortcomings in our study that require comment. A major limitation was the use of single urine sample for estimation of microalbuminuria for logistic reasons. However; this may not change the inferences drawn as most epidemiological studies have followed methodology of single urine sample measurements. Another limitation is its cross-sectional design. Renal involvement is a strong predictor of mortality [54], also in population-based patient samples. Therefore life-time prevalence of renal involvement is much greater than the prevalence found in our cross-sectional survey. Besides, we did not confirm the presence of type 2 diabetes by studying the auto-antibodies, hence it is difficult to believe that our study population consisted of subjects with type 2 diabetes only. Moreover, the current diagnosis of type 2 diabetes according to ADA is fasting plasma glucose (FPG) > = 126 mg/dL. Thus, the previous classification used in the present study (FPG > = 110 mg/dL) permitted the inclusion of patients with impaired glucose tolerance and impaired fasting glucose among the diabetic patients. The presence of retinopathy characteristic of diabetes has been reported in 12.6% of persons with elevated fasting glucose and impaired glucose tolerance and no known history of diabetes, in comparison to 7.9% of nondiabetic persons [55]. The presence of isolated microalbuminuria is also believed to be a biomarker of widespread vascular injury and atherosclerotic burden. In this sense, it does not measure a "kidney disease" per se, but only a secondary and indirect effect of a distant disease process on kidney physiology [56]. Thus, microalbuminuria is more a marker of endothelial dysfunction instead of a marker of renal impairment [57-59]. This fact may also explain why newly diagnosed patients (who probably present other cardiovascular risk factors) presented a rate of microalbuminuria similar to the one observed in the group with known diabetes, while only macroalbuminuria, a more specific marker of renal impairment was significantly different between the two groups.

The strengths of the study are a well-conducted population-based prevalence study, with retinopathy diagnosis based on gold standard fundus photography and comprehensive clinical and biochemical evaluation.

It must be remembered that renal involvement only identifies a group of diabetic patients at high risk of developing complications, including DR, and these patients may benefit from intensive treatment [60]. Identification of renal involvement cannot be used to narrow down the number of patients who need to be screened for DR, as many patients without renal involvement have DR.

## Conclusions

Micro - or macroalbuminuria are highly prevalent in subjects with type 2 diabetes with every sixth individual having some albuminuria. Subjects with micro - and macroalbuminuria are more likely to have DR as those without albuminuria.

## Competing interests

The authors declare that they have no competing interests.

## Authors' contributions

PKR and RR participated in acquisition of data and drafting the manuscript. AG and SSP participated in analysis and writing the manuscript. VK performed the statistical analysis. TS participated in study design and gave final approval of the version to be published. All authors read and approved the final manuscript.
